# Etiology and outcomes of new onset seizure in adult patients: A clinical experience from emergency department of a tertiary care center

**DOI:** 10.12669/pjms.38.5.4411

**Published:** 2022

**Authors:** Noman Ali, Hasnain Abbas Dharamshi, Syed Mustahsan, Suman Noorani

**Affiliations:** 1 Noman Ali, F.C.P.S. (EM). Assistant Professor, Department of Emergency Medicine, Aga Khan University Hospital, Karachi, Pakistan; 2 Hasnain Abbas Dharamshi, M.B.B.S. Resident, Department of Emergency Medicine, Aga Khan University Hospital, Karachi, Pakistan; 3 Syed Mustahsan, MBBS. Resident, Department of Emergency Medicine, Aga Khan University Hospital, Karachi, Pakistan; 4 Suman Noorani, MBBS. Resident, Department of Emergency Medicine, Aga Khan University Hospital, Karachi, Pakistan

**Keywords:** Etiology, New-onset Seizure, Emergency Department,

## Abstract

**Objectives:**

To identify the etiology and outcomes of new onset seizure in adult patients presenting to the Emergency Department (ED), to improve knowledge among healthcare providers regarding diagnosis and hence improve the outcomes.

**Methods:**

This was a descriptive retrospective study conducted at the Emergency Department of Aga Khan University Hospital, Karachi, Pakistan. Adult patients (18 years and above), presented to the ED from January 01, 2019 to June 30, 2020, with new onset seizure were included consecutively. Descriptive data on patient demographics, seizure types, investigations performed, final diagnosis and disposition were collected retrospectively from patient’s file and electronic record. Etiologies of seizure were classified as structural (metabolic, trauma, infection), genetic or idiopathic. The immediate outcomes were reported as hospitalization or discharge from the ED.

**Results:**

In total 198 patients with new onset seizure were included. Majority of the patients were found in middle age group i.e., 35 to 65 years (44.4%). Gender distribution showed slightly higher percentage of females (55.1%). Generalized tonic clonic seizures were found to be the most common type of seizures (74.2%), followed by generalized tonic (12.1%) and focal onset aware seizures (7.5%). Out of total patients no cause was identified in eight patients (4%), whereas in total confirmed causes of new onset seizures, structural lesions of brain were found to be the most common cause (89.6%%), including neurological (23.6%), infectious (4.2%), systemic (13%), metabolic (7%) and toxicological (4%) causes respectively.

**Conclusion:**

This study explores the various etiologies of new onset seizures in adult patients presenting to the ED. The study emphasizes the need for a local guideline for the investigation of new onset seizures in adult patients that would direct emergency physicians in respect of appropriate management, thus to ensure better quality of patient care and outcomes.

## INTRODUCTION

Seizure is a brief alteration in normal electrical brain activity resulting in awareness, perception and behavior changes. There are many unidentified pathological reasons of seizure. Around 8% to 10% of the total population experiences a seizure during their lifetime and only about 2% to 3% of them later develop epilepsy.^1^ Even a single seizure is a frightening and traumatic event with serious potential consequences, information about optimal, evidence-based approach for evaluation of adult patients presenting with seizure is essential.

Seizures are common presentation in the Emergency Department (ED) and account for 1%-2% of all Emergency visits, with 24% representing new onset seizures.^2^ Emergency physician faces many challenges in the evaluation of a patient with new onset seizure because the differential diagnosis is broad and many conditions mimic a seizure. In around 45% of patients with new onset seizure, no cause is identified and less than 10% of patients with new-onset seizures have metabolic or toxicological cause.^3^ The current standard ED management approach for an uncomplicated, first generalized seizure includes performing a history, physical examination that provide the basis for risk stratification and diagnostic testing to exclude seizures provoked by toxic, metabolic, structural and systemic causes.^4^,^5^

Knowledge of common etiology of new onset seizure in adult patient is, therefore, essential for the ED care provider. Data on the etiology and outcomes of new onset seizure that present to the ED is scarce, especially in the low to middle income countries (LMIC). Therefore, we aimed to study and identify the etiology of new onset seizures and its outcomes in the adult patients coming to the ED to improve healthcare provider knowledge in diagnosis and management of these patients.

## METHODS

This was a descriptive retrospective single center study conducted in the Emergency Department of the Aga Khan University hospital (AKUH), Karachi, Pakistan from January 01, 2019 to June 30, 2020. Ethical approval (ERC No# 2020-3553-8832) was obtained from the Ethical Review Committee of the Aga Khan University Hospital on March 16, 2020.

A total of 198 consecutive adult patients (18 years and above), presenting to the ED with new onset of seizure were included in the study. Patients presented with history of trauma were excluded. A structured performa was designed to collect following information through medical chart review i.e. patient demographics, clinical data (signs and symptoms, types of seizures), investigation performed and patient’s disposition. Etiologies of seizure were classified as structural, neurological, infectious, systemic, metabolic and toxicological. The immediate outcomes were reported as patient disposition i.e., hospitalization or discharge.

### Data collection procedure:

Data was collected by two authors. Filters such as fits, jerky movements and seizures were applied to extract the medical record numbers from triage data. Files were reviewed by the data collectors and all adult patients presenting to the ED and found to have new onset of seizure were included. Prior to the initiation of data collection, the data collectors went through a refresher training session to understand the process of AKU Hospital Information Management System (HIMS) to extract files from the medical record and to review patient file and extract relevant information based on questionnaire.

### Data Analysis:

The data was populated and analyzed on SPSS version 20. Descriptive analysis was performed and frequency of continuous variable i.e. age was calculated and categorized into three groups to find the age-specific etiology of new onset seizure. Frequencies and percentages were also calculated for categorical variables i.e. gender, type of seizure, investigations done, diagnosis, disposition and outcomes of patients

## RESULTS

A total of 198 patients were included in the study. The demographic data shows that the number of male and female patients was (44.9%) and (55.1%), respectively. The age was categorized into three groups that is young age (18 to 34 years), middle age (35 to 64 years) and old age (>65 years), of which majority of the patients were in middle age (44.4%) followed by younger age (34%) and old age (21%), respectively. The most common co morbidities were hypertension (34%), diabetes (23.6%) shown in Table-I. Generalized tonic clonic seizures were the most common type of seizure (74.2%), followed by generalized tonic (12.1%) and focal onset aware seizures (7.5%), shown in Table-II. Out of total number of patients only 17 patients (8.6%) had positive family history of seizures. Neuroimaging was performed in 134 patients, of whom 75 patients (55%) showed epileptogenic lesion. Electroencephalogram (EEG) was done in 103 patients and of them 77 patients(74%) had abnormal findings suggestive of seizure activity. Lumbar puncture was performed in 26 patients, of them 10 patients (38%) showed picture of CNS infection.

**Table I:** Demographics, disposition and outcomes of patients

Characteristics	n	%
**Age**		
18 to 34 years	68	34.3
35 to 64 years	88	44.4
≥65 years	42	21.2
**Gender**		
Male	89	44.9
Female	109	55.1
**Comorbids**		
Diabetes	47	23.7
Hypertension	67	33.8
Ischemic Heart Disease	15	7.6
Chronic Kidney Disease	14	7.1
Others	55	27.8
**Disposition from ED**		
Admitted	145	73.2
Discharged	20	20.1
Leave against medical advice LAMA	33	16.7
**Outcomes**		
Discharged from hospital	142	71.7
Expire	03	2.0

**Table II:** Frequencies of Different Types of Seizures.

Types of Seizure	n	%
Generalized Tonic Clonic Seizure	147	74.2
Generalized Tonic Seizure	24	12.1
Focal onset aware seizures	15	7.6
Focal Onset Impaired Awareness Seizures	8	4.0
Myoclonic Seizure	3	1.5
Absence Seizure	1	0.5

Total	198	100.0

Study findings showed that no cause was identified in eight patients (4%) as they left against medical advice from Emergency Department without investigations. Among total confirmed causes of new onset seizures, structural lesions of brain were found to be the most common cause (49%), followed by idiopathic or unknown reason (23.6%), infectious (14.1%), metabolic (9.2%), and toxin related (4.1%) In structural causes of new onset seizures, the most common diagnosis was scar epilepsy (18.4%), followed by space occupying lesion (8.9%) and brain metastasis (3.1%). Idiopathic were 23.6%. Among infectious etiology acute bacterial meningitis was diagnosed as most common cause (4.7%) followed by viral encephalitis (4.2%) and septic encephalopathy (2.6%), cerebral venous sinus thrombosis 4.7% and posterior reversible encephalopathy syndrome 4.7%. Five of the total female patients were pregnant in whom eclampsia was diagnosed as a most common cause of new onset seizure (1.5%). In metabolic causes, uremic encephalopathy was found to be the most common cause (2.6%), followed by hyponatremia (2.1%) and hypoglycemia (1.0%). Final diagnosis of new onset seizure is shown in Table-III. The most common preceding symptom was drowsiness (37%) followed by headache (32%), fever (15%), vomiting (14%) and diarrhea (2%). Present study findings showed that in all age group most common cause of new onset seizure was scar epilepsy. In young age group, meningoencephalitis was found to be the second most common cause while in middle and old age group scar epilepsy was more common

**Table III:** Diagnosis of common etiology of Seizures.

Etiology	Diagnosis	n (%)
Structural	Scar Epilepsy	35 (18.4)
	Space Occupying Lesion (Brain Tumor)	17 (8.9)
	Brain Metastasis	6 (3.1)
	Intra-cranial Bleed	5 (2.6)
	Acute Ischemic Stroke	5 (2.6)
	Intra-cranial Aneurysm	2 (1.0)
	Arterio-venous Malformation	1 (0.5)
	Subarachnoid Hemorrhage	1 (0.5)
	Posterior reversible encephalopathy syndrome	9 (4.7)
	Cerebral venous sinus thrombosis	9 (4.7)
	Autoimmune Encephalitis	2 (1.0)
	Multiple Sclerosis	2 (1.0)
		94 (49%)
Idiopathic	Idiopathic/ Unknown causes	45 (23.6)
		45 (23.6%)
Infectious	Acute bacterial meningitis	9 (4.7)
	Viral encephalitis	8 (4.2)
	Septic Encephalopathy	5 (2.6)
	Dengue Encephalitis	4 (2.1)
	Cerebral Malaria	1 (0.5)
		27 (14.1%)
Metabolic	Uremic Encephalopathy	5 (2.6)
	Hyponatremia	4 (2.1)
	Hypoglycemia	10 (1.0)
	Hypocalcaemia	1 (0.5)
	Hypernatremia	1 (0.5)
	Eclampsia	3 (1.5)
		24 (9.2%)
Drug/Toxin	Alcohol Withdrawal	6 (3.1)
	Drug Overdose	2 (1.0)
		8 (4.1%)

Among total confirmed causes of new onset seizures, structural lesions of brain were found to be the most common cause (38%), followed by neurological (24%), infectious (14%), systemic (13%), metabolic (7%) and toxicological (4%).

In structural causes of new onset seizures, the most common diagnosis was scar epilepsy (18.4%), followed by space occupying lesion (8.9%) and brain metastasis (3.1%). In neurologic causes the most common diagnosis was idiopathic (16.8%) with no certain etiology followed by epilepsy (6.3%). Among infectious etiology acute bacterial meningitis was diagnosed as most common cause (4.7%) followed by viral encephalitis (4.2%) and septic encephalopathy (2.6%). In systemic causes, common etiology was found to be cerebral venous sinus thrombosis (4.7%) and posterior reversible encephalopathy syndrome (4.7%). Five of the total female patients were pregnant in whom eclampsia was diagnosed as a most common cause of new onset seizure (1.5%). In metabolic causes, uremic encephalopathy was found to be the most common cause (2.6%), followed by hyponatremia (2.1%) and hypoglycemia (1.0%). Final diagnosis of new onset seizure is shown in Table-III. The most common preceding symptom was drowsiness (37%) followed by headache (32%), fever (15%), vomiting (14%) and diarrhea (2%). Present study findings showed that in all age group most common cause of new onset seizure was scar epilepsy. In young age group, meningoencephalitis was found to be the second most common cause while in middle and old age group scar epilepsy was more common.

## DISCUSSION

The current study was designed to explore the etiology and outcomes of new onset seizures in patients presenting to the ED of a tertiary care center. The importance of new-onset seizures in adult patients stems from its frequent association with secondary causes. In this study the most common cause of new onset seizure was found to be scar epilepsy followed by idiopathic. This study adds the importance of diagnosing scar epilepsy. Also, we found mostly neurological diagnosis was idiopathic, which needs to be investigated more.

This study adds the importance of diagnosing scar epilepsy; also majority of seizure were found to be idiopathic

In present study most of the patients belong to middle age group. Similar findings are reported in a study done by Chalasani and Kumar, in which 47% of the patients belong to 21 to 40 years of age group.^6^ More studies showed that majority of the patients with new onset seizures belonged to age less than 40 years.^7^

In our study, majority of the patients were females (55.1%). Study done by Bora et al. also found a slight predominance of female patients in his study.^8^ The reason behind female predominance is still not well established but it can be attributed to hormonal changes in females as they are at peak in Middle Ages.

Generalized tonic clonic seizures were the predominant type of seizures in this study. Kanitkar et al. and Ashwin T et al. also reported a higher prevalence of generalized tonic–clonic seizures in adult population.^9^,^10^ Patients with generalized tonic clonic seizures are considered to have a high risk of cognitive sequelae and immediate postictal complications such as aspiration pneumonia.

Neuroimaging including CT scan or MRI brain was performed in most of the patients, but only 55% had abnormal findings. A study done by Ho K, Lawn N on yield of neuroimaging in new onset seizure showed an epileptogenic lesion in 29% of the patients.^11^ EEG was done in 103 patients and of them 77 patients had abnormal findings with very few showing significant epileptic discharges. Similar results were also seen in study done by Hirtz D and Berg A.^12^ EEG showing presence of focal seizures may support in localizing the site involved and may predict seizure recurrence. Normal EEG finding does not rule out the diagnosis of epilepsy.

Structural abnormalities of brain were found to be the most common cause of new onset seizures in the study. In structural abnormalities, scar epilepsy was found to be the most common cause followed by space occupying lesion of brain, cerebral venous sinus thrombosis and posterior reversible encephalopathy syndrome (PRES). Sendil et al. in his study also found stroke as a most common etiology of new onset seizure.^13^ It represents as the most common etiology of epilepsy with an incidence of 2-4% in patients over the age of 60 years.^14^ A systematic review suggested that around 10.6% of the patients presented with seizures are related to intracerebral bleed while up to 8.6% are due to ischemic infarct.^15^

Brain tumors are the second common cause of epilepsy after cerebrovascular accidents in elderly population, accounting for almost 10%–30% of all causes of geriatric epilepsy.^16^ In our study brain tumors are found to be the second most common structural cause of new onset seizures. Around 20%–45% of patients with brain tumors presents with new onset seizures during their course of the disease.^17^ Tumors that are located in the frontal, temporal and parietal lobes are expected to cause seizures compared to tumors located in the medullary region.^18^

Brain metastasis is the third common structural cause of new onset seizure. Overall, 10% of cancer patients develop brain metastasis, but rates may vary, depend upon the type of primary tumor.^19^ Around 20% to 27% of patients with history of brain metastases will experience seizures during their disease course.^20^ There is a higher risk in patients with metastases involving the regions of brain with high epileptogenicity, like motor cortex and temporal lobe. Other risk factors are headache and focal deficit at seizure onset.^21^

In neurologic causes of new onset seizures most common cause was found to be idiopathic (20%) followed by epilepsy (3.5%). In idiopathic cases all investigations including metabolic profile, MRI brain and EEG were normal. Similar results were found in a study done by Kaur et al. in which they didn’t find any cause of new onset seizure in 20% of patients.^22^ Another study done by Pradeep et al. showed that seizures beginning at the age of 20years or more were idiopathic in 44% of patients.^23^

Central nervous system (CNS) infections are the major cause of new-onset seizures and acquired epilepsy in the developing world.^24^ In present study CNS infections are the third most common etiology of new onset seizure in which meningoencephalitis was the most common cause (63%).The risk of seizures may vary depending upon the cause of encephalitis. In viral encephalitis more than 40% of patients develop seizures.^25^ Septic encephalopathy was found to be the second most common infectious etiology of new onset seizure. Up to 70% of patients with sepsis have some degree of encephalopathy.^25^ Both bacterial and viral infections can cause encephalopathy with symptoms frequently include seizures. It could be due to an excessive immune response to infection.^25^ Dengue encephalitis is another common infectious etiology of seizure in our study. Dengue infection can cause multisystem disorder. There are several neurologic manifestations that have been reported, including Guillain-Barré syndrome, acute disseminated encephalomyelitis, and myositis, however, the most widely reported is encephalopathy. It occurs due to direct infiltration of neurons by the dengue virus.^25^

In systemic causes of new onset seizure CVST and PRES were the most common. In 12% to 31.9% of patients with CVST, seizures are the common presenting feature. Up to 44.3% of patients with CVST can develop seizures in the early course of the disease. In patients with CVST, especially with raised intracranial pressure, seizures may adversely affect the prognosis. Study done by Singh TD et al. showed that mortality in patients with CVST who develop seizures was three times higher than in those without seizures.^25^

Posterior reversible encephalopathy syndrome (PRES) is a clinical and radiological syndrome, commonly associated with acute severe hypertension. It was diagnosed in 4.7% of the patients. PRES is commonly associated with symptoms including headache, altered consciousness, visual disturbances and seizures

In present study 7% of the patients with new onset seizure were diagnosed with metabolic abnormality. Most common cause was uremic encephalopathy (38.4%) followed by hyponatremia (30.7%). Uremic encephalopathy occurs due to renal insufficiency resulting in production of toxic metabolites and electrolyte abnormalities. Around one third of patients with uremic encephalopathy develop seizures. A study done by Singh TD et al. showed metabolic abnormalities causing 12% of the cases of new onset seizures, in whom hyponatremia was the most common cause.^25^ Adults with secondary seizures were diagnosed most often with head injury (25.2%), brain tumors (19.9%) and stroke (18.4%) as causative factors in a study conducted by Wajid J et al.^26^ Among total number of cases, 8.6% have positive family history of epilepsy while a study conducted in Aziz H et al. showed that family history was positive up to 32 %.^27^

Of the total confirmed causes of new onset seizure 4% were due to toxicological causes. Out of eight patients six patients had seizure due to alcohol withdrawal and two patients had due to drug over dose. Alcohol withdrawal seizures can occur early as six hours after the cessation of alcohol ingestion. Ninety percent occurs within 48 hours.^25^ It is a diagnosis of exclusion. About one third of patients with alcohol withdrawal seizures develop delirium tremens, with the seizure terminating before the development of seizure. About one third of patients with alcohol withdrawal, only 1-3% develops delirium tremens.

### Limitations of the study:

The current study is a single center retrospective study. The study is subject to certain inherent limitations and potential biases, including missing or incomplete information of patients going against medical advice. Participating site may not be the representative of whole population, and patients with new onset seizures likely differ from region.

## CONCLUSION

To our knowledge this is the first study related to etiology and outcomes of adult patients presented with new onset seizure to the Emergency Department in Karachi, Pakistan. Diagnosis of seizures, classification of type of seizure and determination of possible causes require appropriate selection of diagnostic tools including CT scan MRIs, EEG, blood chemistry, and lumbar puncture. The study emphasizes the need for local guidelines regarding the investigation of new onset seizures in adult patients. Local guidelines would direct our Emergency Physicians in respect of proper investigations, thus to ensure better quality patient care and outcomes.

### Authors’ Contribution:

**NA:** Conceived, designed and did statistical analysis & editing of manuscript.

**HAD & SN:** Did data collection and manuscript writing and final editing of manuscript.

**SM & SN:** Did statistical analysis as well as data collection.

**HAD** taken the responsibility and is accountable for all aspects of the work in ensuring that questions related to the accuracy or integrity of any part of the work are appropriately investigated and resolved.
